# Zinc and Silver-Infused Calcium Silicate Cement: Unveiling Physicochemical Properties and In Vitro Biocompatibility

**DOI:** 10.7759/cureus.48243

**Published:** 2023-11-03

**Authors:** Meghan Singh, Chitra Shivalingam, Sheron Blessy, Saravanan Sekaran, Keerthi Sasanka, Dhanraj Ganapathy

**Affiliations:** 1 Prosthodontics, Saveetha Dental College and Hospitals, Saveetha Institute of Medical and Technical Sciences, Saveetha University, Chennai, IND

**Keywords:** characterization, health care, regenerative properties, biocompatibility, bioactive materials, calcium silicate cement

## Abstract

Introduction: Calcium silicate-based types of cement have gained recognition in various dental applications due to their exceptional sealing capabilities, bioactivity, and minimal adaptability. However, these materials have certain shortcomings that can lead to mechanical failures and premature degradation. The inclusion of metal ions into their structure is expected to promote their biological activity. This article focuses on the preparation and characterization of calcium silicate cement to enhance its fundamental material properties, by introducing zinc and silver while retaining its biomaterial characteristics.

Aim: This study aims to evaluate the biomedical potential of zinc and silver-impregnated bioactive calcium silicate cement.

Materials and methods: The calcium silicate powder was synthesized via the sol-gel method. Tetraethyl orthosilicate, calcium nitrate, silver nitrate, and zinc nitrate were sequentially added to create the bioactive calcium silicate material. The synthesized particles underwent physicochemical characterization using techniques such as scanning electron microscopy, X-ray diffraction, Raman spectroscopy, and biological characterization through in vitro hemocompatibility assays.

Results: The study's results revealed the presence of multiple crystalline phases (Ag_6_Si_2_O_7_, Zn_2_SiO_4_, CaCO_3_) as indicated by X-ray diffraction. Raman spectra displayed vibrations associated with Si-O-Si and Zn-O bonding in the zinc and silver-infused bioactive calcium silicate. Scanning electron microscopy confirmed a mixture of spherical and sheet-like morphologies, while energy dispersive spectra confirmed the presence of elements Ca, Si, Zn, Ag, O, and C. In vitro hemocompatibility testing affirmed the material's biocompatible nature.

Conclusion: In conclusion, the zinc and silver-infused calcium silicate cement was successfully synthesized through an in-house procedure and demonstrated biocompatibility. The inclusion of zinc and silver, known for their osteogenic and antimicrobial properties, is anticipated to enhance the cement's biological properties and broaden its utility in dentistry. Further in vitro and in vivo investigations are imperative to validate its clinical applications and elucidate the molecular mechanisms underlying its efficacy.

## Introduction

The use of regenerative endodontic techniques in clinical endodontics has significantly expanded over the past two decades, despite their rapid evolution. These techniques have been considered valuable adjuncts to traditional methods like root canal treatment or apexification when dealing with necrotic, immature permanent teeth with a poor prognosis [1].

Biomaterials and biomaterial-based scaffolds have emerged as promising tools in regenerative endodontic procedures and have made significant strides as part of the biological triad of tissue engineering [2]. These biomaterials can be utilized for essential pulp treatments, temporary intracanal medications, definitive fillings, apical procedures, and regenerative techniques, enhancing dental treatment approaches across various domains. Specifically, dental materials with a calcium silicate foundation have gained prominence in endodontics due to their proximity to bone, connective tissue, and dental tissues. Understanding their characteristics is essential as they play a crucial role in their interaction with biological tissues and their ability to promote biomineralization processes. Previous studies have demonstrated that the controlled release of silicate into the microenvironment can enhance cellular adhesion and subsequently stimulate osteogenic differentiation of bone marrow mesenchymal stem cells [3,4].

Recent advancements in bone tissue engineering have focused on developing load-bearing biodegradable implants for temporary use, leveraging the high osseointegration properties and bioactivity of calcium silicate cements [5,6]. However, challenges remain, such as their high dissolution rate in biological contexts, which can impact long-term clinical performance, especially in patients with osteoporosis. Additionally, the relative instability of natural biomaterials can lead to mechanical malfunction and premature deterioration. Therefore, various surface modification techniques have been employed to improve their biological and chemical stability.

The field of materials science, in collaboration with biomedical sciences, has enabled the development of biomaterials aimed at regenerating or replacing damaged bone tissue [7]. Several studies have explored the addition of dopants like zinc and silver ions to bioactive coatings, finding that the right quantities can positively influence cellular responses while excessive concentrations may hinder cell growth [8-10]. For load-bearing implant applications, metals are typically preferred. The concept of creating load-bearing, biodegradable implants for temporary use has recently gained attention as a potential solution to address various issues associated with current implants [11]. These biodegradable implants safely dissolve in the physiological environment due to chemical breakdown without posing unusual health risks [12]. Biocomposites for orthopedic implant applications have been developed using ball milling and sintered zinc and calcium silicate powders [13,14]. The synthesis of Zn-substituted calcium silicate coatings has garnered interest for its excellent anticorrosion properties and potential increased biocompatibility [15]. These coatings exhibit exceptional biological activity, leading to a high rate of osteoblast proliferation and corrosion resistance, ultimately enhancing wound healing following implantation [16-19]. Next to zinc, silver has emerged as a promising antibacterial material to prevent bone infections, thanks to its advantages, including rapid antibacterial action, high efficiency, and reduced susceptibility to bacterial resistance. Silver has demonstrated effectiveness against Enterococcus, a predominant bacterium in chronic root canal infections that can lead to therapy failure [20,21]. However, the use of silver-containing biomaterials poses challenges due to silver's significant cytotoxicity, which can trigger inflammatory responses, oxidative stress, and hinder tissue regeneration.

Bioactive calcium silicates are well-known materials for biomedical applications [22]. Impregnating zinc and silver could induce changes in the host material, rapidly influencing biological properties. The intention of this study is to investigate the effects of silver and zinc on the physicochemical properties of calcium silicate and biocompatibility. The reason for choosing zinc and silver is owing to their bifunctional roles in osteogenesis and antimicrobial properties. In this study, we fabricated zinc and silver-infused bioactive calcium silicate and conducted a comprehensive analysis of its basic structural and morphological properties, as well as its biocompatibility. A significant challenge exists in incorporating two or more metal ions into the calcium silicate structure. Therefore, the current study is aimed at developing calcium silicate incorporated with dual metal ions and investigating the physicochemical properties and biocompatibility of the synthesized biomaterial.

## Materials and methods

Materials

All the chemicals and reagents used in this study were of analytical grade and were employed without further purification. Tetraethyl orthosilicate was obtained from Alfa Aesar (Haverhill, USA), while nitric acid was purchased from Spectrum Reagents and Chemicals Pvt. Ltd. (Ernakulam, India). Calcium nitrate was acquired from Merck & Co. (Rahway, USA), and silver nitrate and zinc nitrate were obtained from Sisco Research Laboratories Pvt. Ltd (Mumbai, India).

Synthesis protocol

Calcium silicate was synthesized using the sol-gel method developed in-house with the following composition: SiO (0.3 M), CaO (0.3 M), ZnO (0.1 M), and AgO (0.1 M). The preparation of calcium silicate bioactive materials began with the dissolution of tetraethyl orthosilicate (1.37 mL) in a mixture of ethanol (5 mL) and double-distilled water (10 mL), with the addition of nitric acid (3 mL) as a catalyst to expedite the gelation process. After the formation of a complete gel, calcium nitrate (0.708 g) was dissolved in 10 mL of double-distilled water and added to the silicate matrix. Similarly, zinc nitrate (0.298 g) and silver nitrate (0.17 g) were separately dissolved in double-distilled water and introduced into the base material with an hour time gap. The fabricated sol was dried in a hot air oven at 100°C for 24 hours, followed by thermal treatment at 600°C to stabilize the materials.

Characterization techniques

The synthesized calcium silicate was characterized to analyze its properties. X-ray diffraction patterns were used to assess the crystalline phases with Cu Kα wavelength (Bruker D8 advance, Bruker Corporation, Billerica, USA). Functional group properties were analyzed through Raman spectroscopy (WITEC ALPHA300 RA - Confocal Raman Microscope with AFM, WITec, Ulm, Germany). Morphological and elemental analysis was conducted using JEOL (JSM-IT 800, JEOL, Ltd., Akishima, Japan) and Oxford Instrumentation, respectively.

In vitro hemocompatibility assessment

Erythrocyte compatibility testing was performed to evaluate the biocompatibility of the bioactive materials with blood cells [23]. Blood was collected from a healthy volunteer and treated with the anticoagulant ethylenediaminetetraacetic acid to prevent coagulation. Red blood cells (RBCs) were then separated by centrifugation at 4°C for 10 minutes and washed three times with phosphate-buffered saline (PBS, pH 7.4) to remove plasma and other blood components. Hemocompatibility assays were conducted to assess the erythrocyte rupture rate (RBC lysis behavior) in the presence of bioactive materials, which was compared with negative and positive controls (10% Triton X). All bioactive material concentrations were analyzed in triplicate (n = 3). The test samples were incubated at 37°C for one hour. Afterward, erythrocytes incubated with the bioactive materials were centrifuged, and the RBC rupture rate was measured at a wavelength of 540 nm. To perform a blood compatibility assessment, ethical approval was received from the Saveetha Dental College and Hospitals Human Ethical Committee Clearance Board (IHEC/SDC/FACULTY/23/PROSTHO/209).

## Results

X-Ray diffraction pattern

**Figure 1:**
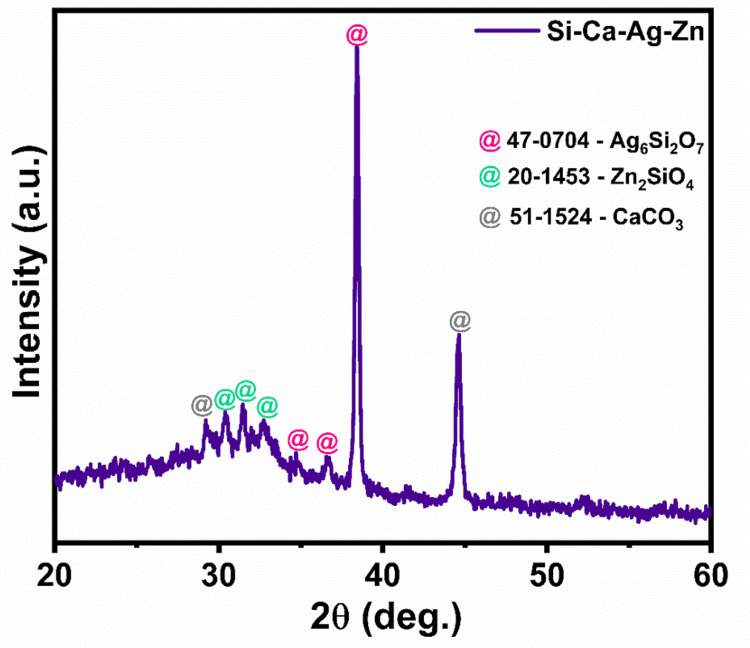
X-ray diffraction patterns of synthesized zinc and silver-infused bioactive calcium silicate

To analyze the crystal structure of zinc and silver-infused bioactive calcium silicate, we conducted an X-ray diffraction study. Figure 1 illustrates the semi-crystalline nature of calcium silicate, as evident from the multiple crystalline phases observed in the XRD patterns. The diffracted peaks provide authentication of mineral formation, indicating the presence of Ag_6_Si_2_O_7_, Zn_2_SiO_4_, and CaCO_3_ within the synthesized calcium silicate material.

From the X-ray powder diffraction (XRD) patterns, it becomes evident that silver and zinc are effectively bonded with the silica matrix. Additionally, the crystalline patterns of calcium carbonate were observed, likely influenced by environmental carbonate [24]. This XRD pattern demonstrates the successful incorporation of silver and zinc into the calcium silicate silica glass matrix.

Raman spectroscopy

Raman spectroscopy serves as a crucial tool for identifying the vibrational modes of oxides that contain essential metal ions. It provides a distinctive fingerprint for the molecules, highlighting their structural and functional groups.

**Figure 2:**
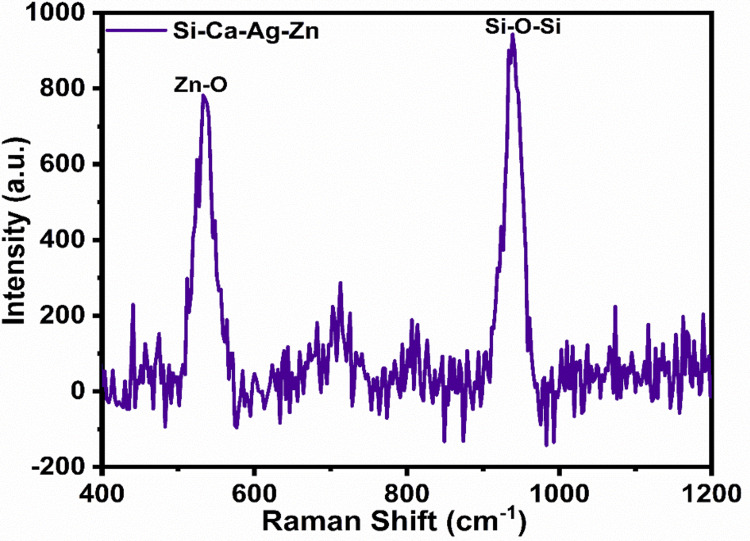
Raman spectra for zinc and silver-infused bioactive calcium silicate

Figure 2 reveals the presence of Si-O-Si bonding and Zn-O bonding in the Raman spectra, confirming the formation of the materials. It's important to note that due to the semi-crystalline nature, some peaks may exhibit minor noise.

Morphological and elemental analysis

**Figure 3:**
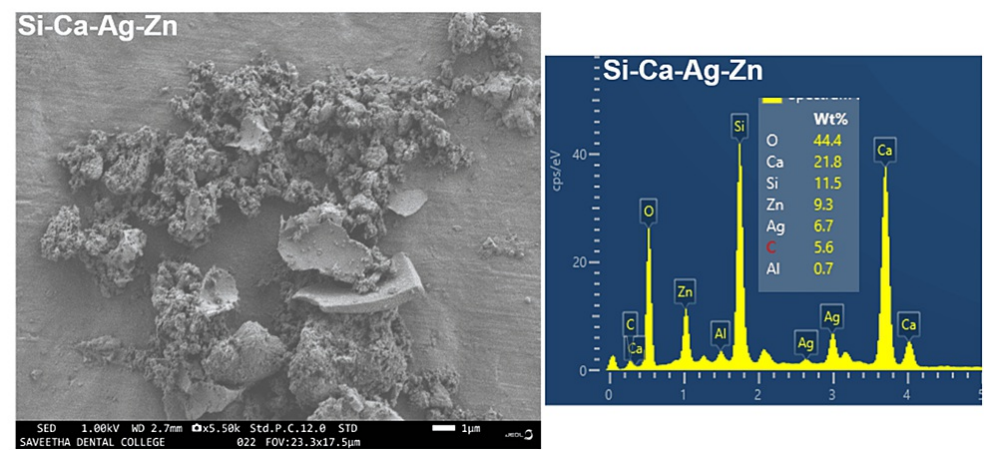
Morphological and elemental analysis of synthesized zinc and silver-infused bioactive calcium silicate

For the analysis of morphological characteristics and elemental properties of the fabricated calcium silicate-based material, FE-SEM (Field Emission Scanning Electron Microscopy) was employed for morphology, and EDS (Energy Dispersive Spectroscopy) was utilized for elemental analysis. In Figure 3, we elucidate the surface morphology and elemental composition of the synthesized zinc and silver-infused bioactive calcium silicate. It is apparent that the zinc and silver-infused bioactive calcium silicate exhibits a morphology characterized by small spherical structures and aggregated spheres integrated onto a sheet-like surface. The EDS spectra provide elemental distribution data for relevant components, including Ca, Si, Zn, Ag, O, and C peaks, further confirming the formation of the materials.

Hemocompatibility assessment

Blood compatibility serves as a crucial parameter for evaluating the interaction between synthesized bioactive materials and red blood corpuscles, particularly in the context of regenerative applications.

**Figure 4:**
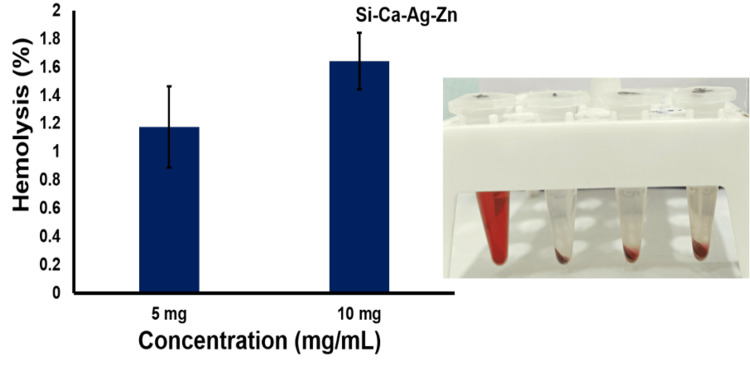
Hemocompatibility assessment of synthesized zinc and silver-infused bioactive calcium silicate

Following the American Society for Testing and Materials (ASTM) standard - F756 (Standard Practice for Assessment of Hemolytic Properties of Materials), it is considered acceptable for materials to exhibit up to 5% lysis. Figure 4 illustrates that the zinc and silver-infused bioactive calcium silicates demonstrate minimal lysis, measuring below 1.6% lysis at a concentration of 10 mg/ml. This level of hemolysis is well within the acceptable range for biomedical applications, affirming the compatibility of this material.

## Discussion

Endodontic biomaterials have found versatile applications in essential pulp treatments, temporary intracanal medications, definitive fillings, apical operations, and regenerative procedures. Their utilization has significantly improved dental treatment strategies across various domains. Specifically, dental materials with a calcium silicate base have gained prominence in endodontics due to their proximity to bone, connective tissue, and dental tissues. The material's inherent characteristics play a crucial role in its interaction with biological tissues and its ability to promote biomineralization processes.

In our study, we successfully produced zinc and silver-impregnated bioactive calcium silicate using the sol-gel technique. We proceeded to analyze the material's structural, morphological, and biocompatible properties. The study employed various analytical methods to gain insights into the material's properties. X-ray diffraction analysis, a nondestructive method, precisely determined the crystallographic structure, chemical composition, and physical characteristics of the material. The analysis revealed the presence of relevant mineral phases, including Ag_6_Si_2_O_7_, Zn_2_SiO_4_, and CaCO_3_. Raman spectroscopy, another valuable analytical method, assessed the vibrational energy modes of the material through scattered light. In our study, Raman spectra for zinc and silver-infused bioactive calcium silicate displayed vibrational modes corresponding to Si-O-Si and Zn-O bonding.

For microstructural examination of the silver and zinc-impregnated calcium silicate material, we employed FE-SEM. Additionally, we conducted elemental analysis and chemical characterization using EDS. These techniques revealed a small spherical and sheet-like morphology with relevant elemental composition, including Ca, Si, Zn, Ag, O, and C peaks in the EDS spectra. To assess the material's blood compatibility, we conducted hemocompatibility assessments. The objective was to enhance testing methods for evaluating material-mediated hemolysis based on the ASTM F756-17 standard. Our findings indicated significant blood compatibility within the acceptable range, suggesting the material's suitability for biomedical applications.

Calcium silicate biomaterials hold promise for a wide range of biomedical applications, including the regeneration of both soft and hard tissues. These materials enhance the development of various cell types, including osteoblasts, fibroblasts, odontoblasts, cementoblasts, pulp cells, and various stem cells [25]. Moreover, they trigger the chemical development of calcium phosphate or apatite layers when submerged in biological fluids, expanding their therapeutic potential.

The ideal bone cement should possess handling qualities that allow for minimally invasive administration and maintain integrity during implantation. Reduced osteogenesis and impaired bone tissue repair and regeneration in infected sites remain significant clinical concerns [26]. In our study, we developed a process for synthesizing bioactive calcium silicates infused with zinc and silver to address these issues. Our research aims to address the limitations of existing materials, which can prematurely deteriorate and fail mechanically. By engineering calcium silicates to improve fundamental material properties, we have created a novel zinc and silver-impregnated bioactive calcium silicate cement. This approach optimally preserves the physicochemical properties and retains biocompatibility. The material can be further tested for antimicrobial activity and osteogenic properties by comparing the inclusion of individual ions and combinations into calcium silicate cement using appropriate in vitro models.

Limitations

The study primarily conducted in vitro experiments and assessments. While these provide valuable insights into the material's properties and compatibility, they may not fully replicate the complex in vivo conditions, such as the dynamic interactions with host tissues, immune responses, and other biological factors. The study predominantly focuses on short-term evaluations of material properties and compatibility. Long-term assessments, which are crucial for assessing the durability and performance of biomaterials in clinical settings, were not addressed. Addressing these limitations in future studies will provide a more comprehensive understanding of the potential applications and challenges associated with zinc and silver-impregnated bioactive calcium silicate biomaterials in clinical practice.

## Conclusions

The synthesis of the zinc and silver-infused bioactive calcium silicate cement was conducted using an in-house procedure and found to not interfere in the material properties and biocompatibility. The research mainly conducted short-term evaluations of the zinc and silver-infused bioactive calcium silicate cement. Long-term investigations are essential to ascertain the material's durability and effectiveness over extended periods. While the study demonstrated the material's biocompatibility, it did not delve into the underlying biological mechanisms that govern its interaction with the host tissues. Future studies should aim to provide a more comprehensive understanding of these mechanisms.
